# “The Standard Procedure” for Investigation of Oral Neutrophils in Oral Diseases

**DOI:** 10.1155/2023/1308326

**Published:** 2023-04-27

**Authors:** Peter Østrup Jensen, Pernille Dukanovic Rikvold, Kristine Røn Larsen, Mette Rose Jørgensen, Camilla Kragelund

**Affiliations:** ^1^Costerton Biofilm Center, Department of Immunology and Microbiology, University of Copenhagen, Copenhagen, Denmark; ^2^Department of Clinical Microbiology, Copenhagen University Hospital (Rigshospitalet), Copenhagen, Denmark; ^3^Center for Rheumatology and Spine Diseases, Institute for Inflammation Research, Copenhagen University Hospital (Rigshospitalet), Copenhagen, Denmark; ^4^Department of Odontology, Oral Pathology & Medicine, Faculty of Health and Medical Sciences, University of Copenhagen, Copenhagen, Denmark

## Abstract

**Aim:**

There is need of an objective “standard procedure” that is reliable and clinically applicable for estimating oral neutrophil content in relation to oral diseases.

**Methods:**

Forty-one patients with suspected oral candidosis (OC) and nine healthy controls with no oral mucosal disease were flushing with 10 ml mouth rinse (MR) (sterile phosphate-buffered saline) for 1 min. Aliquots were stored on different conditions to explore stability, storage, and fixation conditions for analysis by flow cytometry.

**Results:**

The optimal storage and fixation condition for MR was by fixation 1 : 1 in 10% formalin and stored at 5°C. This procedure yielded stable results up to 7 days after collection. The ability of the optimized method to relate oral neutrophils to inflammation was demonstrated by the significantly higher number of neutrophils in patients with primary OC (*p* = 0.0334) compared to healthy controls.

**Conclusion:**

This method is rapid, reliable, and clinically applicable for establishing the content of oral neutrophils. We demonstrate increased density of oral neutrophils in the MR of patients with OC. The potential of the method is to be “the standard procedure” for investigation of the oral inflammation in patients with oral diseases as it is noninvasive and provides high stability, clinical relevance, and minimal handling.

## 1. Introduction

Approximately, 50% of healthy individuals carry *Candida* as part of the oral microflora and the most frequent species is *Candida albicans* [1]. *Candida* species are opportunistic pathogens as they, aside from being part of the normal human microbiota, can cause oral infection under certain conditions [2]. In particular, the interaction between the host and the fungal microflora can go from symbiosis to dysbiosis with selective overgrowth of *Candida* species [3]. Oral candidosis (OC) can be primary OC when the infection arises without presence of a preexisting oral disease and secondary OC is when related to an oral disease, e.g., oral lichen planus (OLP), geographic tongue (GT), dry mouth, or salivary hypofunction. The diagnosis of OC is established on the patient's symptoms or clinical signs of OC and paraclinical findings of *Candida* infection in cytosmears, culture, or biopsies [4]. The histopathologic pattern of chronic OC is hyperparakeratinization of the oral lesion with embedded hyphae and presence of microabscesses of neutrophils and a chronic inflammatory infiltrate subjacent in the connective tissue [5]. Oral neutrophils may accumulate in the oral cavity via migration though the oral mucosal epithelium and the gingival crevice [6]. The density of oral neutrophils is increased in mouth rinse (MR) from patients with periodontal disease with positive associations between density and disease severity [7, 8]. This increased accumulation likely results from the participation of neutrophils in the inflammatory response to dysbiotic periodontal microbiota establishing bacterial dental plaques below the gum line [9]. Neutrophils activate several antimicrobial mechanisms including phagocytosis, production of reactive oxygen species, release of proinflammatory substances, proteolytic enzymes, and antimicrobial peptides and the formation of neutrophil extracellular traps [10, 11]. In accordance, flow cytometrical analysis of the neutrophils in MR has made it possible to further relate the activation and function of oral neutrophils to periodontal disease [12]. Thus, oral neutrophils in MR have only been used in research of periodontitis, probably because the clinical use of oral neutrophils in MR awaits procedures adapted to the limited time and restricted capacity for sample handling during examination and treatment in clinics.

In OC, the accumulation of neutrophils in the oral mucosa is mediated by interleukin-17 (IL-17) from T-17 helper cells of the adaptive immune response [13] as well as by IL-17 secreted by innate lymphoid cells from the innate immune response [14]. The oral neutrophils found in saliva mainly originates from the gingival crevice [6], but we expect the MR procedure to allow collection of oral neutrophils by loosening neutrophils attached in the oral mucosa.

Until now, however, there has not been a standardized method to access the oral neutrophil density in oral diseases. Thus, the objective of this study was to develop a reliable and reproducible method with minimal handling and minimal effect of the duration of storage for determination of the oral neutrophil density that allows comparison of progression, stagnation, and response to treatment of oral diseases. This objective was approached by comparing the impact of easy fixation and simple storage conditions prior to further processing in committed laboratories on the density of neutrophils in MR determined by flow cytometry. The clinical relevance of the selected method was evaluated by comparing the density of neutrophils in MR from patients with conditions associated with the presence of oral neutrophils such as patients with OC.

## 2. Material and Methods

### 2.1. Participants

For establishment of the method, one group consisting of seven healthy volunteers without mucosal diseases or OC were recruited from the Department of Clinical Microbiology, University Hospital Copenhagen, Denmark.

For evaluating the clinical feasibility of the established method, all patients consulting the Clinic of Oral Medicine at the Department of Odontology at the University of Copenhagen in May 2019 with suspicion of OC and dental students with GT were included in the study. As healthy control, nine students were included. Dental and medical history together with clinical and histopathological diagnoses of oral diseases were obtained from the patient's dental record the same day as MR was obtained at the Department of Odontology. Participants were not instructed or examined differently than they normally are before a consultation at the Clinic of Oral Medicine. Thus, patients had to refrain from brushing their teeth, drinking, eating, or smoking less than 1 hr before the consultation. Just as there is no systematic assessment of periodontal disease, why periodontal diseases were not evaluated. The Ethical Committee of Copenhagen approved the study as part of a method development study protocol number: H-4-2012-FSP at the Section of Oral Pathology & Medicine.

### 2.2. Mouth Rinse Sampling

All participants rinsed with 10 ml of sterile phosphate-buffered saline (PBS) (pH 7.4) MR for 1 min, where after the samples were collected in a 50 ml centrifuge tube (VWR®) [15] and kept on ice (0°C) until further handling in the laboratory ∼1 hr after sampling at which time the temperature in the sample was 3°C. All samples were collected between 8:30 and 10:30 AM.

### 2.3. Candida Diagnostics

Cytosmears were taken from areas with symptoms or clinical signs of OC and patients were diagnosed with OC when one or more *Candida* hyphae were found in a cytosmear and diagnosed without OC when no *Candida* hyphae were identified [16].

### 2.4. Study Designs

#### 2.4.1. Development of the Method

For the development of the method, MR was collected from seven healthy volunteers. The tubes containing the MR were thoroughly vortexed for 30 s whereafter 1 ml of each MR was transferred to four smaller centrifuge tubes with the purpose of comparing four different fixation and storage conditions. Two of the centrifuge tubes were added 1 : 1 PBS and left unfixed and two were fixated in 1 : 1 with 10% formalin. In a preliminary pilot-study focusing on fixation we found formalin to be superior to methanol and ethanol (data not shown). One of each were stored at room temperature (RT) (+24°C) or at 5°C as confirmed by internal thermometers in our refrigerators.

MR was collected from all patients consulting the Clinic of Oral Medicine with suspected OC (no other criteria). When arriving to the laboratory the MR was fixated in 1 : 1 with 10% formalin and stored at 5°C until preparation for flow cytometry.

#### 2.4.2. Flow Cytometry of Oral Neutrophils

The MR preparations were vortexed and 100 *µ*l were transferred to BD Trucount™ tubes (BD 340334; Becton Dickinson). Five microliters of fluorescein isothiocyanate-labelled CD15 antibodies (5554019, BD Bioscience, La Jolla, CA, USA) and 5 *μ*l of allophyocyanin-labelled CD45 antibodies (555485) were added to each tube and the tubes were placed on ice and incubated for 30 min in the dark. Processing of the samples was completed by addition of 0.9 ml 1x fluorescence activated cell sorter (FACS) lysis solution (349202, BD Bioscience). The samples were kept on ice in the dark and analyzed immediately after preparation for flow cytometry. A flow cytometer (BD FACSCanto™, BD Bioscience) was used to measure and analyze the samples. The instrument was equipped with a 15 mV argon ion laser tuned at 488 nm, and a red diode laser emitting at 635 nm for excitation. Light-scatter and fluorescence parameters from at least 10,000 events were recorded. Leukocytes were discriminated by gating on CD45 and the neutrophils were further discriminated by gating on size and morphology and on CD15. The instrument was calibrated using BD™ Cytometer Setup and Tracking Beads (642412, BD Bioscience). We have previously used the same antibody combination to quantitate exudated human neutrophils [17–19] and the specificity for the leukocyte subtypes has been verified in peripheral human blood where the identity of leukocyte subtypes was validated according to the morphology as determined by the light scatter. Gating of the fluorescence and the light scattering was set according to similarly stained human peripheral blood samples. The concentration of neutrophils was calculated from the ratio of recorded neutrophils and counting beads as previously described [17–19].

### 2.5. Statistical Analysis

GraphPad Prism, version 9.0.0. (GraphPad Software Inc., La Jolla, CA) was used for descriptive analysis and statistics. For “stability of oral MR”, the data were normalized by logarithmic transformation before analyses with two-way ANOVA test with Tukey's multiple comparisons correction. The groups of clinical patients were compared using Mann–Whitney *U* test with Dunn's multiple comparisons correction and *F* test. Two-sided *p* < 0.05 was considered statistically significant.

## 3. Results

**Figure 1:**
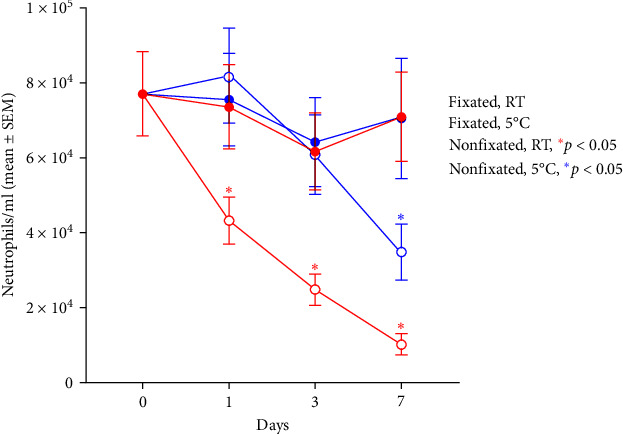
Effect of fixation, storage temperature, and duration of storage on the concentration of neutrophils in mouth rinse. The mouth rinses were either fixated in 10% formalin or left unfixed and stored at 5°C or room temperature (RT) before preparation and flow cytometrical analysis on day 0, 1, 3, and 7 after collection. Statistically significant decline in number of neutrophils per milliliter in nonfixed samples despite of storage temperature when comparing to day 0. Statistical analysis by two-way ANOVA.  ^*∗*^*p* < 0.05.

The impact of fixation in 10% formalin, storage temperature, and duration of storage on the measured concentration of neutrophils is shown in Figure 1. A significantly decreased concentration of neutrophils was recorded at day 1, 3, and 7 in nonfixated samples stored at RT (*p* < 0.0001) and at day 7 in nonfixated samples stored at 5°C (*p* < 0.0001). In contrast, the concentration of neutrophils was not changed significantly in the fixated samples stored at RT or at 5°C indicating that the concentration of neutrophils remains stable in samples when fixated in 10% formalin for at least 7 days.

**Figure 2:**
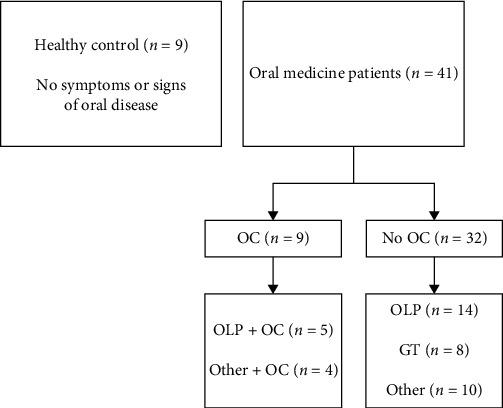
Subgroups of the 50 participants in the clinical study. GT, geographic tongue; OC, oral candidosis; OLP, oral lichen planus; Other, other diagnoses.

The distribution of participants in the clinical study is illustrated in Figure 2. Nine healthy controls who had no oral symptoms or oral diseases were included in the study thirty-five patients (Gender: 23 females and 12 males. Median age in years (range): 57 (12–83)) and six students with GT (no data on gender or age) were included in the study during May 2019. The most frequent diagnosis was OLP (*n* = 19) followed by GT (*n* = 9). Other diagnoses included mucosal hyperplasia (*n* = 4), leukoplakia (*n* = 3), ulceration (*n* = 3), suspicion of primary OC (*n* = 2), dry mouth (*n* = 1), and hyposalivation due to radiotherapy (*n* = 1). Nine patients were diagnosed with chronic OC. Two patients with primary OC and seven with secondary OC; in relation to OLP (*n* = 5), leukoplakia (*n* = 1), and radiotherapy associated hyposalivation (*n* = 1).

The concentration of oral neutrophils was significantly higher in all patients diagnosed with OC (mean: 2,16,467 neutrophils/ml, range: 50,900–5,41,000) compared to the healthy volunteers (mean: 61,802 neutrophils/ml, range: 8,720–1,41,000) (*p* = 0.0334). There was no statistical difference between the OC group and the group without OC (no OC) (mean: 1,66,506 neutrophils/ml, range: 5,850–1,510,000) (*p* = 0.1539), and between the healthy volunteers and the no OC group (*p* = 0.6641). Within the group of healthy controls, the variation was smaller than within the two patient subgroups (*F* test, *p* < 0.009).

## 4. Discussion

The purpose of comparing fixation and storage conditions was to investigate the durability of MR samples in relation to storage of samples before analysis. Storage of samples is relevant in clinical settings where analysis immediately after sample collection often is not possible. In our study, we were able to prevent a significant decrease of the density of oral neutrophils by fixating the samples in a final concentration of 10% formalin as we found no significant difference in the density of oral neutrophils between day 0 and fixated samples stored up to day 7 at RT (*p* = 0.9994) or at 5°C (*p* = 0.9988). If storage for beyond 7 days is needed, we advise to examine the stability of samples exposed to extended storage. Storage at 5°C or at RT appeared to have no significant effect on the estimation of the density of oral neutrophils, but we prefer storing the samples at 5°C since the storage temperature is frequently available and to avoid varying temperatures associated with storage at RT. Ten percent formalin was chosen as this concentration belongs to the fixatives often available at the clinical setting as it has long been used for preservation of clinical samples, e.g. biopsy specimens [4, 20].

**Figure 3:**
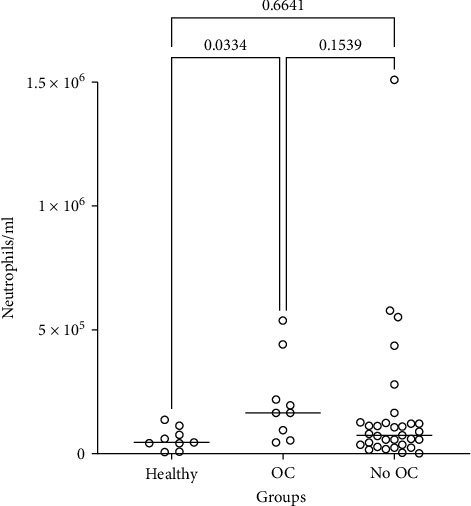
The concentration of neutrophils in mouth rinse in patients with oral candidosis (OC) (mean = 216467; SD = 169377) or without OC (no OC) (mean = 166506; SD = 284084) and healthy controls (mean = 61802; SD = 43880). Statistically significant increased number of neutrophils per milliliter in patients diagnosed with OC when compared to the healthy controls. Bars indicate medians. Statistical analysis by Mann–Whitney *U* test.

To evaluate the clinical relevance of our optimized method we compared the density of oral neutrophils in MR from clinical patients and healthy controls. Significant difference was found in the concentration of oral neutrophil load between patients diagnosed with primary OC or with lichenoid manifestations and secondary OC and healthy controls. The lack of information on the distribution of the density of neutrophils in MR from OC, prevented reliable calculations of the appropriate sample sizes in advance. However post hoc calculation suggests a power of 0.5, which indicates that our study has been underpowered and that the number of persons in the groups should be increased. To guide structuring future examinations with appropriate power, we propose to include the means and SD of the groups displayed in the legends to Figure 3. OC is an infection that causes inflammation of the oral mucosa with manifestations of neutrophil microabscesses [5]. These neutrophil microabscesses may be a source of the increased neutrophil load in MR from patients with OC. We found no indication for increased density of neutrophils in MR from patients with OLP without OC, which is in-line with the dominance of lymphocytes in the chronical inflammatory infiltrate of the oral mucosa in lichenoid manifestations [21]. We did not find higher neutrophil density in MR from participants with GT, which we had expected as neutrophils are found accumulated superficially in the epithelium at the edge of the GT lesions [22].

The association between increased density of neutrophils in MR and the severity of periodontitis has long been recognized [7]. In fact, in the most severe stages of periodontitis the density of neutrophils in MR may be more than fivefold higher than the density of neutrophils in MR from healthy controls [7, 8]. In the present study, however, the density of neutrophils in MR from the OC group was approximately threefold higher than the density of neutrophils in MR from healthy controls indicating that the total accumulation of oral neutrophils is more pronounced in severe stages of periodontitis than in OC. However, the local density of oral neutrophils may be higher in the microabscesses accompanying OC [5].

The density of neutrophils in MR from the OC group was only significantly higher compared to the healthy group (*p* = 0.0334), but not when comparing to the no OC patients (*p* = 0.1539), supporting the potential of assessing oral neutrophils densities for monitoring of oral inflammatory diseases, e.g., inflammatory bowel diseases. In addition to inflammatory diseases and infections, oral neutrophil counts may also be an easily accessible biomarker for assessing tumor progression and prognosis, as well as an indicator of the treatment outcome of bone marrow transplantation following immunosuppressive therapy in patients with non-Hodgkin lymphoma or multiple myeloma [23].

Limitations of the sampling were revealed by initial experiments where the density of neutrophils in MR varied throughout the day (not shown). For that reason, all samples were collected at the same time span (between 8 and 9:30AM in the morning). The larger variation within the two patient subgroups suggests that not only oral diseases but also systemic diseases, medication, and lifestyle factors influence the oral neutrophil load. Thus, when quantifying oral neutrophils in patients to determine their level of oral inflammation a thorough clinical examination is needed to diagnose all diseases of the oral mucosa, periodontium, jawbone, and salivary glands as several diseases will affect the oral neutrophil concentration. Also, a detailed medical record is needed focusing on systemic (extraoral) conditions and medication. Likewise, standardized behavior guidelines before sampling of MR regarding oral function should be considered in order to ensure no direct exogen interferences, e.g., food and beverage, oral hygiene products, and lifestyle factors. In particular, this MR method seems relevant when evaluation oral neutrophils in relation to treatment response in relation to both oral and systemic disease, medication, and inflammation.

In conclusion, we have developed a noninvasive, user-friendly enumeration of oral neutrophils with the capability to indicate increased accumulation of oral neutrophils in OC compared to healthy subjects. Future investigations that include more participants and an extended storage period are necessary to conclude on the durability of fixed samples. The method would benefit from usage of a more eco-friendly and less health hazardous fixative than 10% formalin.

## Data Availability

The data used to support the findings of this study are available from the corresponding author upon request.
